# Effects of senolytic drugs on human mesenchymal stromal cells

**DOI:** 10.1186/s13287-018-0857-6

**Published:** 2018-04-18

**Authors:** Clara Grezella, Eduardo Fernandez-Rebollo, Julia Franzen, Mónica Sofia Ventura Ferreira, Fabian Beier, Wolfgang Wagner

**Affiliations:** 1 Helmholtz-Institute for Biomedical Engineering, Stem Cell Biology and Cellular Engineering, RWTH Aachen University Medical School, Pauwelsstraße 20, 52074 Aachen, Germany; 2 Department for Hematology, Oncology, Hemostaseology and Stem Cell Transplantation, Faculty of Medicine, RWTH Aachen University, Aachen, Germany

**Keywords:** Senolytic drugs, Senescence, Mesenchymal stromal cells, DNA methylation, Telomere attrition, ABT-263, Quercetin, Danazol, Nicotinamide riboside

## Abstract

**Background:**

Senolytic drugs are thought to target senescent cells and might thereby rejuvenate tissues. In fact, such compounds were suggested to increase health and lifespan in various murine aging models. So far, effects of senolytic drugs have not been analysed during replicative senescence of human mesenchymal stromal cells (MSCs).

**Methods:**

In this study, we tested four potentially senolytic drugs: ABT-263 (navitoclax), quercetin, nicotinamide riboside, and danazol. The effects of these compounds were analysed during long-term expansion of MSCs, until replicative senescence. Furthermore, we determined the effect on molecular markers for replicative senescence, such as senescence-associated beta-galactosidase staining (SA-β-gal), telomere attrition, and senescence-associated DNA methylation changes.

**Results:**

Co-culture experiments of fluorescently labelled early and late passages revealed that particularly ABT-263 had a significant but moderate senolytic effect. This was in line with reduced SA-β-gal staining in senescent MSCs upon treatment with ABT-263. However, none of the drugs had significant effects on the maximum number of population doublings, telomere length, or epigenetic senescence predictions.

**Conclusions:**

Of the four tested drugs, only ABT-263 revealed a senolytic effect in human MSCs—and even treatment with this compound did not rejuvenate MSCs with regard to telomere length or epigenetic senescence signature. It will be important to identify more potent senolytic drugs to meet the high hopes for regenerative medicine.

**Electronic supplementary material:**

The online version of this article (10.1186/s13287-018-0857-6) contains supplementary material, which is available to authorized users.

## Introduction

Senolytic drugs hold the perspective to specifically target senescent cells and thereby to rejuvenate tissues or organisms [1]*.* Several compounds have been suggested to possess senolytic effects, including navitoclax (ABT-263) [2], quercetin [1], danazol [3], and nicotinamide riboside [4]. ABT-263 inhibits BCL-2 protein family members, which are crucial regulators of the apoptosis pathway [2, 5, 6]. ABT-263 was shown to deplete senescent cells of human umbilical vein epithelial cells (HUVECs), IMR90 human lung fibroblasts, and murine embryonic fibroblasts, but not human primary pre-adipocytes [5]. Danazol is a synthetic androgen with telomere elongating capacity, which has been used to target accelerated telomere attrition—a hallmark of aging and senescence [3]. Quercetin is a proteasome activator with anti-oxidant properties [7] that triggers apoptosis via the BCL-2 pathway [1]. Nicotinamide riboside increases levels of nicotinamide adenine dinucleotide (NAD^+^). Aged mice supplemented with nicotinamide riboside revealed increased lifespan and rejuvenated muscle stem cells [4].

Primary cells undergo a limited number of divisions before entering the state of replicative senescence. The process of senescence induces changes in morphology, metabolism, secretory phenotype, and differentiation potential of cells, thereby having a significant impact on experimental outcomes and affecting their therapeutic potential [8]. This applies particularly to mesenchymal stromal cells (MSCs), which raise high hopes in tissue engineering and are concurrently tested in a multitude of clinical trials [9]. MSCs comprise a multipotent subset of cells, capable of differentiation towards osteogenic, chondrogenic, and adipogenic lineages. The selective removal of senescent MSCs from cultures might improve standardization and effectiveness of cell preparations for cell therapeutics in regenerative medicine. We have therefore directly compared the senolytic capacity of ABT-263, quercetin, danazol, and nicotinamide riboside in human MSCs during long-term culture.

## Methods

### Cell culture

Mesenchymal stromal cells were isolated from the femoral bone marrow of three donors after orthopaedic surgery. All samples were taken after informed and written consent and the study was approved by the ethics committee of RWTH Aachen University Medical School (permit number: EK300/13). Culture medium consisted of Dulbecco’s modified Eagle medium (DMEM, 1 g L^−1^ glucose; PAA, Pasching, Austria) supplemented with 1% penicillin/streptomycin (PAA), 1% l-glutamine, 10% pooled human platelet lysate that was generated as described previously [10], and 0.1% heparin (5000 lU ml^−1^; Ratiopharm, Ulm, Germany). Cells were cultured at 37 °C, in an atmosphere containing 5% CO_2_, and passaged by trypsinization when they reached 90% confluency with a re-seeding density of 10,000 cells cm^−2^. The immunophenotype and three-lineage differentiation potential of MSCs was validated as described previously [10, 11].

### Senolytic compounds

Cells were treated either with ABT-263 (Selleck Chemicals, Houston, TX, USA), quercetin (Sigma-Aldrich, St. Louis, MO, USA), nicotinamide riboside (ChromaDex, Irvine, CA, USA), or danazol (Sigma-Aldrich). These compounds were dissolved in DMSO (VWR, Radnor, PA, USA) (ABT-263), ethanol (VWR) (quercetin and danazol), or phosphate-buffered saline (Sigma-Aldrich) (nicotinamide riboside), according to the manufacturer’s instructions, and subsequently diluted in cell culture medium to working concentrations of 10 μM (ABT-263), 100 μM (quercetin and danazol), or 20 mM (nicotinamide riboside). If not stated otherwise, the compounds were applied only for 3 days.

### Viability assays

Viability was measured with the ATPlite Kit (PerkinElmer, Waltham, MA, USA) according to the manufacturer’s instructions. In addition, viability was measured with the AlamarBlue™ Cell Viability Reagent (ThermoFisher Scientific, Waltham, MA, USA) and the results were always consistent.

### Co-culture of senescent and non-senescent cells

Mesenchymal stromal cells at early (passage 3) and late (before proliferation arrest) passages of the same donor were simultaneously taken into culture and stained with the PKH67 Green Fluorescent Cell Linker Kit and PKH26 Red Fluorescent Cell Linker Kit, respectively (both Sigma-Aldrich). These cells were subsequently mixed at a 1:1 ratio and treated with each of the four drugs for 1 day. Fluorescence was subsequently analysed by fluorescence microscopy (EVOS; Life Technologies GmbH, Darmstadt, Germany) or quantified on a FACS Canto II (BD, Heidelberg, Germany).

### Senescence-associated beta-galactosidase staining

This assay was performed with the Senescence Detection Kit (Abcam, Cambridge, UK) according to the manufacturer’s instructions.

### Telomere length measurement

Genomic DNA was isolated with the NucleoSpin® Tissue kit (Macherey-Nagel, Düren, Germany). Telomere length was determined by monochrome multiplex quantitative PCR as described previously [12]. In brief, C_T_ values of telomere qPCR were compared to the corresponding C_T_ values of a single-copy gene qPCR of the same sample. The resulting T/S ratio correlates with the number of telomeric repeats.

### Epigenetic senescence signature

Genomic DNA was isolated as already described and bisulfite converted with the EZ DNA Methylation™ Kit (Zymo Research, Irvine, CA, USA). DNA methylation was analysed by pyrosequencing at six senescence-associated CG dinucleotides (SA-CpG sites) related to the genes *GRM7*, *CASP14*, *CASR*, *SELP*, *PRAMEF2*, and *KRTAP13-3*, as described previously [13]. In brief, bisulfite-converted DNA was analysed on a PyroMark Q96 ID System (Qiagen, Hilden, Germany) and results were evaluated with the PyroMark CpG SW 1.0 software (Qiagen). The DNA methylation values were then implemented into linear regression models to predict passage numbers [14].

### qRT-PCR analysis

RNA was isolated via a NucleoSpin RNA extraction kit (Macherey-Nagel) and analysed with a NanoDrop ND-1000 spectrophotometer (Thermo Scientific, Waltham, MA, USA). Semi-quantitative qRT-PCR was conducted with *Power SYBR* green master mix (Applied Biosystems, Foster City, CA, USA) and the following primers: *β-actin*, forward GGCACCACACCTTCTACAAT and reverse AACATGATCTGGGTCATCTTCTC; *p16*, forward GGTCGGGTAGAGGAGGT and reverse ATCATCATGACCTGGATCGG; *IL-6*, forward AGACAGCCACTCACCTCTT and reverse ACTCTTGTTACATGTCTCCTTTCTC; *PAI-1*, forward CTGGTGAATGCCCTCTACTTC and reverse GGCGTGGTGAACTCAGTATAG. Results were normalized to β-actin.

### Statistics

All experiments were performed with three independent biological replicas and the results are presented as mean ± standard deviation (SD). Statistical significance was estimated by two-tailed paired Student’s *t* test.

## Results

**Fig. 1:**
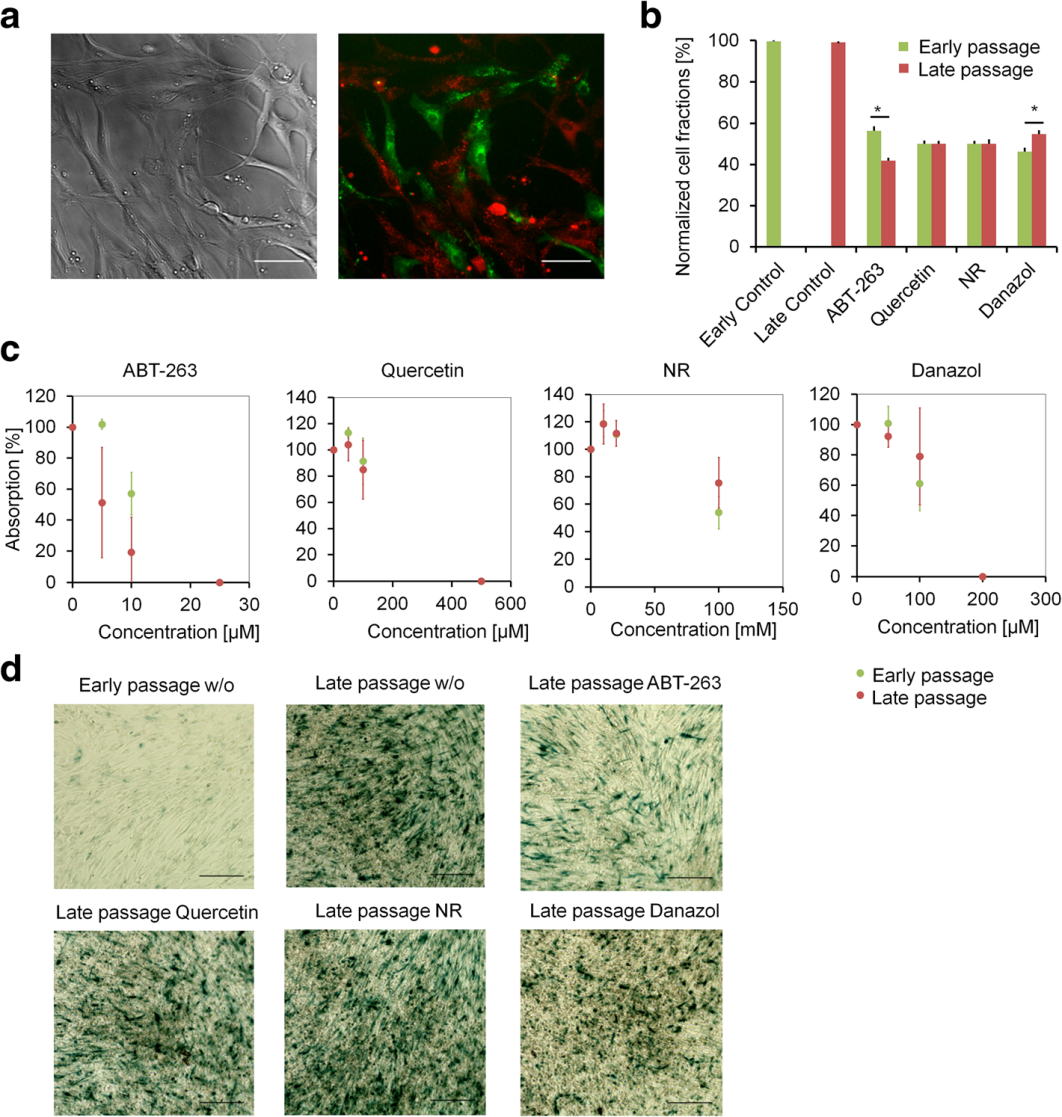
ABT-263 has senolytic effects in human MSCs. **a** Exemplary phase-contrast and fluorescence microscopic images of non-senescent cells (PKH67, green, passage 3) and senescent cells (PKH26, red, passage 12). w/o = without additional drug;  Scale bars = 100 μm. **b** Normalized fractions of cells determined by flow cytometric quantification of red (senescent) and green (non-senescent) cells indicate that particularly ABT-263 reduced senescent cells (normalized to untreated controls, *n* = 3, mean ± SD; *p ≤ 0.05). **c** Dose–response curves analysed after 3 days of treatment with drugs and viability estimated by flow-cytometric assays (normalized to untreated controls, *n* = 3, mean ± SD; individually assessed for early and late passage cells). **d** SA-β-gal staining performed either in non-senescent (passage 3) or senescent (passage 12) MSCs upon treatment with senolytic drugs for 3 days (exemplary images depicted, scale bars = 200 μm). NR nicotinamide riboside

In order to evaluate the specificity of these four different senolytic drugs we first tested the half-maximal effective concentration (EC50) for human MSCs (see Additional file 1: Figure S1). Based on that, we determined working concentrations for subsequent experiments: 10 μM for ABT-263, 100 μM for quercetin, 100 μM for danazol, and 20 mM for nicotinamide riboside. Similar concentrations have been used previously for other cell types [1, 4, 5, 15]. However, it needs to be taken into account that the relatively high concentration of DMSO, which is required as solvent for ABT-263, can already exert toxic effects (see Additional file 1: Figure S2). To address whether the potentially senolytic drugs target specifically senescent MSCs we conceived a simple co-culture experiment: MSCs at early passages (passage 3) were labelled with PKH67 in green and corresponding cells at late passages (at proliferation arrest) were labelled with PKH26 in red. These cells were mixed 1:1, treated with the drugs for 1 day, and then analysed by fluorescence microscopy and flow cytometry (Fig. 1a, b; three biological replicas). ABT-263 treatment significantly reduced the proportion of senescent MSCs (*p* = 0.018; 8.1% less senescent cells), although the specificity for senescent cells was relatively low. In contrast, quercetin and nicotinamide riboside did not affect the ratio of early and late passage cells and danazol even slightly increased the proportion of late passage cells (*p* = 0.037; 4.7% more senescent cells). Alternatively, we analysed dose–response curves in MSCs either at early or late passages (*n* = 3). These results support the notion that the toxic effect of ABT-263 was more pronounced on late passage cells (Fig. 1c). Furthermore, staining of senescence-associated beta-galactosidase (SA-β-gal) in MSCs at late passages was particularly reduced upon treatment with ABT-263 (Fig. 1d). Taken together, ABT-263 showed a mild senolytic effect in MSCs.

**Fig. 2:**
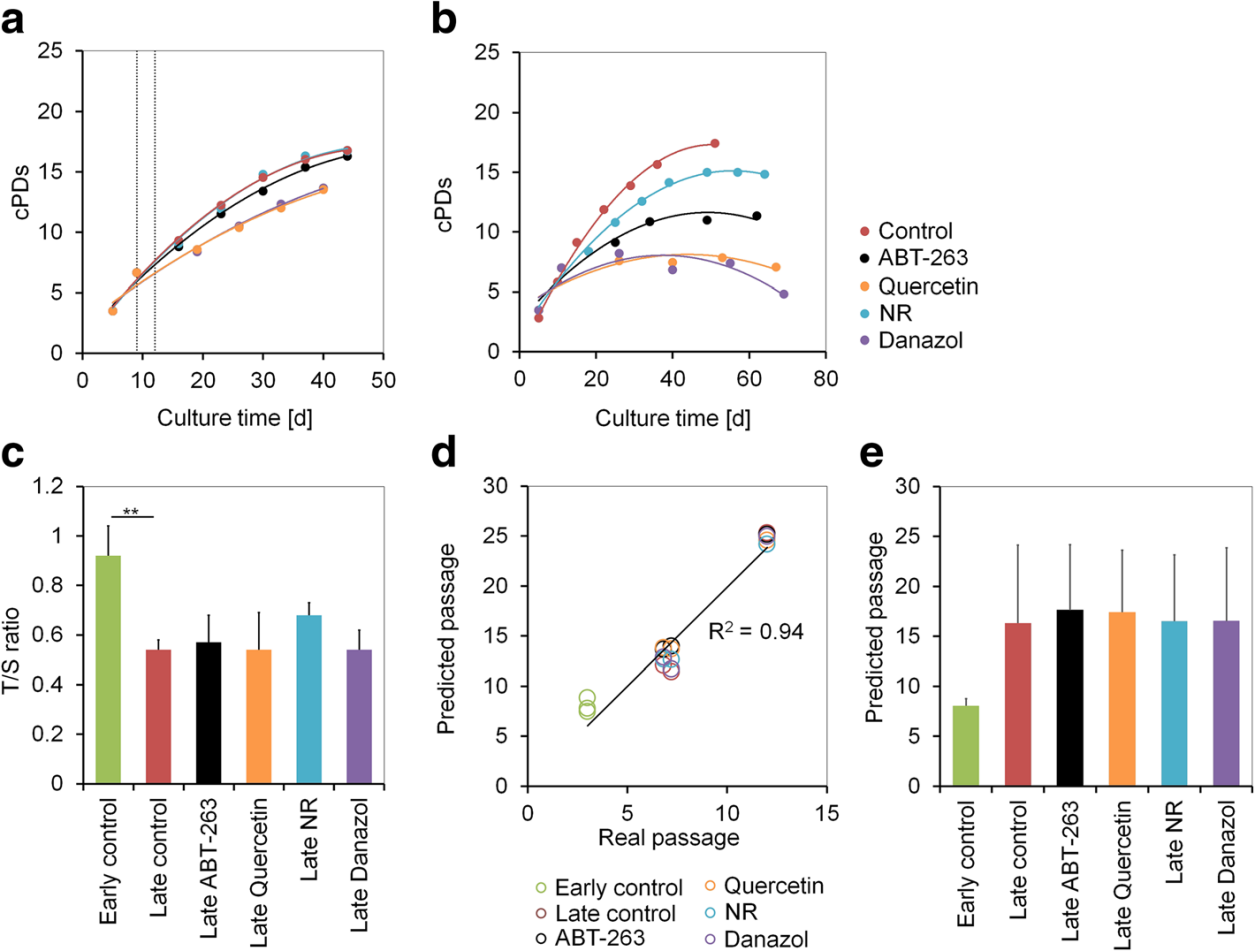
Treatment with senolytic drugs did not support long-term expansion or affect molecular markers of senescence. Long-term growth curves of cumulative population doublings (cPDs) **a** after initial pulse treatment for 3 days (d; period indicated by dashed lines) or **b** with continuous treatment with drugs (results exemplarily depicted for one donor). **c** Relative telomere length measured as telomere to single copy gene (T/S) ratio (monochrome multiplex qPCR) of non-senescent and senescent MSCs with and without senolytic drug treatment (*n* = 3, mean ± SD; **p ≤ 0.01). **d** Comparison of real and predicted passage numbers in non-senescent and senescent MSCs with and without senolytic drug treatment. **e** Average number of predicted passages directly compared for the different treatments (*n* = 3, mean ± SD). NR nicotinamide riboside

Removal of senescent cells might eliminate paracrine factors that trigger growth arrest [16] and thereby delay replicative senescence during culture expansion. To evaluate the effects on long-term growth curves of MSCs we supplemented each of the four drugs for either 3 days at passage 3 or during the entire culture period. Upon short-term treatment with quercetin and danazol, the maximal number of cumulative population doublings (cPDs) declined, probably due to the toxic effects, whereas nicotinamide riboside and ABT-263 did not impact on culture expansion (Fig. 2a; *n* = 3). For long-term treatment we applied 50% reduced concentrations of the drugs and all of them accelerated the proliferation arrest (Fig. 2b; *n* = 3). Thus, none of these potentially senolytic drugs prolonged the culture expansion of MSCs.

Subsequently, we analysed the effect of the four senolytic drugs on molecular markers of senescence. Expression of the senescence-associated genes cyclin-dependent kinase inhibitor 2A (*CDKN2A* or *p16*), interleukin 6 (*IL-6*), and plasminogen activator inhibitor-1 (*PAI-1*) increased at later passages and was even slightly increased upon treatment with senolytic drugs (see Additional file 1: Figure S3). Telomere length was estimated by ratios in monochrome multiplex quantitative PCR, and showed a significantly decline in untreated MSCs during culture expansion (*p* = 0.006) [12]. Yet none of the senolytic drugs increased the average telomere length at later passages (Fig. 2c). Alternatively, we analysed the effect of the senolytic drugs on senescence-associated epigenetic modifications. DNA methylation changes at specific CpG sites are acquired in a highly reproducible manner during replicative senescence of MSCs. We have previously demonstrated that such epigenetic changes provide a robust surrogate marker to estimate the state of replicative senescence [13]. In fact, the results of our six CpG epigenetic senescence signature revealed good correlation of predicted and real passage numbers (*R*^2^ = 0.94), although passage numbers were generally over-estimated. Notably, none of the tested drugs had significant impact on the senescence-associated DNA methylation changes (Fig. 2d, e).

## Discussion

Recently, senolytic drugs raised high hopes for regenerative medicine, aging, and age-related diseases [1]. Quercetin has previously been demonstrated to be effective against senescent human endothelial cells and mouse MSCs [1]; ABT-263 was shown to specifically target senescent human HUVECs or fibroblasts [5]; nicotinamide riboside and danazol have been demonstrated to rejuvenate senescent stem cells [3, 4]. However, none of these compounds have been systematically analysed in human MSCs.

In this study, ABT-263 revealed moderate selectivity for senescent MSCs but also affected non-senescent cells, as described previously [17]. For quercetin, danazol, and nicotinamide riboside we did not observe any senolytic effect. Furthermore, none of the investigated drugs had a positive effect on culture expansion, telomere length, or epigenetic rejuvenation. The discrepancy from the aforementioned reports might be due to the different cell type used in this study. Thus, there is a need to identify more potent senolytic compounds for human MSCs. Recently some additional candidates were described [18, 19] and it is also conceivable that combinations of different drugs could better reduce the burden of senescent cells with less side effects. We used relatively high concentrations of the drugs as we anticipated that hereby the senolytic effect would be more pronounced. On the other hand, lower concentrations would allow reduction of toxic solvents and facilitate discerning the impact on survival and proliferation. Other cell cycle regulators, such as CDKN2B (p27) and the retinoblastoma proteins (RB1, RB2/P130, and P107), are well known to be functionally relevant for senescence of human MSCs [20]. Furthermore, senescence of MSCs is associated with several other changes—for example, in the secretome, autophagy, and metabolism—and these aspects should also be analysed. In addition, it might be conceivable that a senolytic compound modifies the senescence-associated secretory phenotype (SASP) without directly rejuvenating the cells. However, none of the investigated drugs had any positive effect on either the key cytokine IL-6 or PAI-1, which was shown to be part of crucial SASP regulation pathways [21]. The concept of senolytic drugs is attractive, but further research is required to specifically target the senescent cells within MSC preparations.

## Additional file

Additional file 1: **Figure S1.** Dose–response curves for EC50 determination. **Figure S2.** Dose–response curves of solvent controls. **Figure S3.** Expression analysis of senescence-associated genes. (PDF 1049 kb)

## Abbreviations

cPD: Cumulative population doubling
DMEM: Dulbecco’s modified Eagle medium
EC50: Half-maximal effective concentration
HUVEC: Human umbilical vein epithelial cell
MSC: Human mesenchymal stromal cell
SA-β-gal: Senescence-associated beta-galactosidase staining
SD: Standard deviation
