# Supplementary material for: Effects of senolytic drugs on human mesenchymal stromal cells
Source: Stem Cell Res Ther. 2018 Apr 18;9:108. doi: 10.1186/s13287-018-0857-6 (PMC5907463; doi:10.1186/s13287-018-0857-6)
Supplement: Supplementary file 1 — Figure S1. Dose–response curves for EC50 determination. Figure S2. Dose–response curves of solvent controls. Figure S3. Expression analysis of senescence-associated genes. (PDF 1049 kb) [file 13287_2018_857_MOESM1_ESM.pdf]

Additional file 1

Effects of Senolytic Drugs on Human Mesenchymal Stromal Cells

Clara Grezella, Eduardo Fernandez-Rebollo, Julia Franzen, Mónica Sofia Ventura Ferreira, Fabian Beier, Wolfgang Wagner

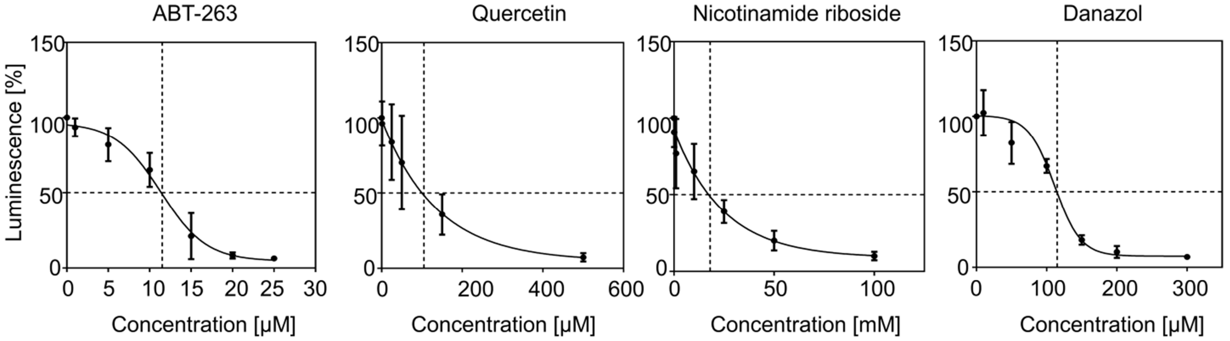

Supplemental Figure 1: Dose response curves for EC50 determination. Viability assay for MSCs at passage 5. Graphical determination of EC50 values is indicated by dashed lines. n = 3.

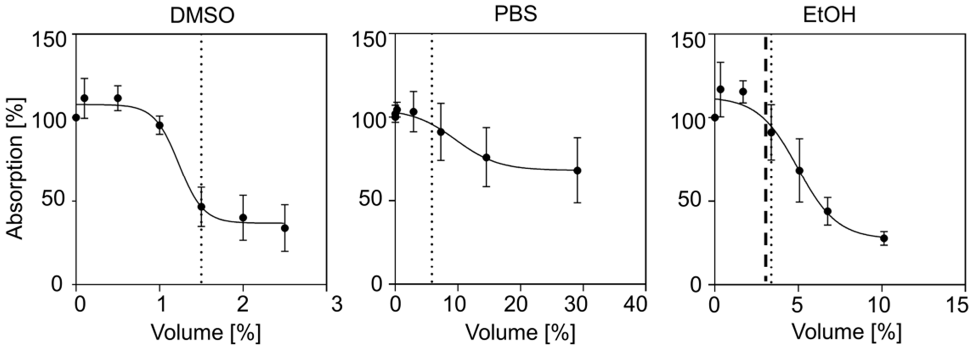

Supplemental figure 2: Dose response curves of solvent controls. Absorbance based AlamarBlue™ Cell Viability assay for MSCs that were treated with DMSO (solvent for ABT-263), PBS (solvent for NR), and ethanol (solvent for quercetin and danazol); n = 3. Working concentrations of solvents are indicated by lines (for EtOH: dashed line quercetin, pointed line danazol).Direct comparison with the results of figure S1 is hampered by variation between different cell preparations and viability assays. Either way, the results clearly demonstrate that particularly DMSO, which is used as solvent for ABT-263, exerts toxic effects at the working concentrations – albeit the cells could still be culture expanded - and this need to be taken into account.

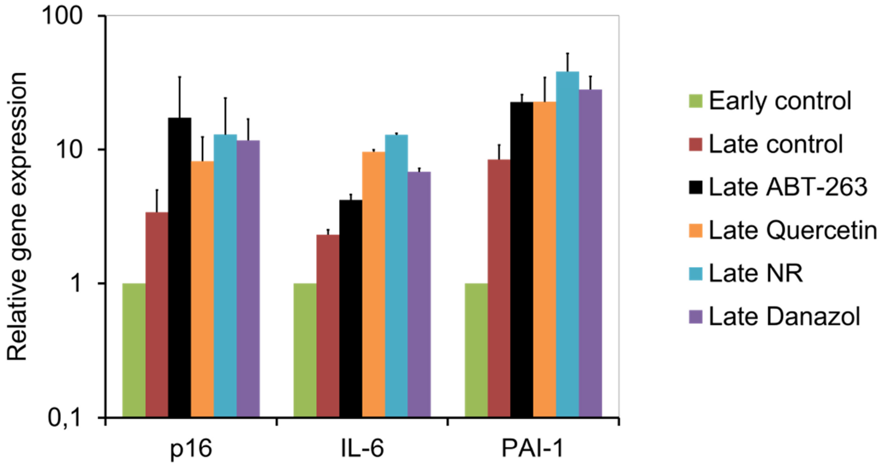

Supplemental figure 3: Expression analysis of senescence associated genes. Relative mRNA expression of cyclin-dependent kinase inhibitor 2A (p16), interleukin 6 (IL-6), and plasminogen activator inhibitor-1 (PAI-1) was analyzed by semiquantitative RT-PCR (results were normalized to early passage controls, n = 3, mean ± SD). As expected, these genes were higher expressed in controls of later passage. However, upon treatment with senolytic drugs gene expression was in tendency even higher, but this did not reach statistical significance.
